# Comparative transcriptome analysis of different tissues of *Hylomecon japonica* provides new insights into the biosynthesis pathway of triterpenoid saponins

**DOI:** 10.3389/fbinf.2025.1625145

**Published:** 2025-07-07

**Authors:** Bing He, Teng Xu, Shaowei Xu, Huqiang Fang, Qingshan Yang

**Affiliations:** ^1^ College of Pharmacy, Anhui University of Chinese Medicine, Hefei, China; ^2^ Key Laboratory of Xin’an Medicine, Ministry of Education, Anhui University of Chinese Medicine, Hefei, China

**Keywords:** Hylomecon japonica, transcriptome sequencing, triterpenoid saponins, squalene synthase, differentially expressed genes

## Abstract

Triterpenoid saponins are one of the main activities of roots and rhizomes of *Hylomecon japonica*, with various pharmacological activities such as antibacterial, anticancer, and anti-inflammatory. To elucidate the biosynthesis pathway of triterpenoid saponins in *H. japonica*, DNA nanoball sequencing technology was used to analyze the transcriptome of leaves, roots, and stems of *H. japonica*. Out of a total of 99,404 unigenes, 78,989 unigenes were annotated by seven major databases; 49 unigenes encoded 11 key enzymes in the biosynthesis pathway of triterpenoid saponins. Nine transcription factors were found to be involved in the metabolism of terpenoids and polyketides in *H*. *japonica* and a spatial structure model of squalene synthase in triterpenoid saponin biosynthesis was established. This study greatly enriched the transcriptome data of *H. japonica*, which is helpful for further analysis of the functions and regulatory mechanisms of key enzymes in the biosynthesis pathway of triterpenoid saponins.

## 1 Introduction

*Hylomecon japonica* (Thunb.) Prantl and Kündig is a perennial herb of the Hylomecon in the family Papaveraceae. Its roots and rhizomes are its main medicinal parts, which are enriched with active ingredients such as alkaloids (Suo and Zhang, 2013), saponins (Ma, 2022), phenols (Feng, 2019), and flavonoids (Lee et al., 2012). It has various pharmacological activities such as anti-inflammatory (Chen et al., 2023), antibacterial (Choi et al., 2010), and anti-tumor (Chae et al., 2012) effects. Among them, triterpenoid saponins such as Hylomeconoside A and Hylomeconoside B exhibit cytotoxicity towards human gastric cancer MGC-803 cells and human promyelocytic acute leukemia cell line HL-60, and are the main components exerting anti-tumor effects (Qu et al., 2017).

The biosynthesis of triterpenoid saponins mainly consists of three parts (Xu et al., 2021). In the initial stage, the upstream substances 3-isopentenyl pyrophosphate (IPP) and dimethylallyl pyrophosphate (DMAPP) were synthesized by the mevalonic acid (MVA) pathway and the methylerythritol phosphate (MEP) pathway. During the terpenoid skeleton construction stage, IPP and DMAPP were catalyzed to form 2,3-oxidized squalene under the action of geranyl diphosphate synthase (GPPS), farnesyl pyrophosphate synthase, squalene synthase (SS), and squalene cyclooxygenase (SQLE). During the modification stage, 2,3-oxidized squalene was undergoesed structural modifications such as cyclization, hydroxylation, and glycosylation by β - aromatic resin synthase (β-AS), cytochrome P450 (CYP450), and glycosyltransferase (UGT) to synthesize triterpenoid saponins. Xu P. et al. (2024) elucidated the complete biosynthetic pathway of astragaloside IV, the main active triterpenoid saponin of *Astragalus membranaceus*, and reconstructed this biosynthetic mechanism in *Nicotiana benthamiana* to allow heterologous production of astragaloside IV. Transcriptome sequencing of the stems, roots, flowers, and leaves of Akebia trifoliata revealed that in the comparative group of stems, roots, flowers, and leaves, DEGs related to triterpenoid saponin synthesis were mainly enriched in terpenoid skeleton biosynthesis, and the highest number of up-regulated DEGs were observed in the stems. These up-regulated genes may be associated with higher medicinal value in stems of Akebia trifoliata (Qian et al., 2022).

RNA sequencing (RNA-seq) is characterized by high-throughput and high-precision datasets, which refine research results down to single nucleotides and can detect the overall transcriptional activity of any species. Compared with traditional chip platforms, the unique advantage of transcriptome sequencing is that it does not require pre-designed probes for known sequences (Li et al., 2013). At present, RNA-seq has been applied in the study of the biological characteristics and biosynthesis of various Chinese herbal medicines such as *Clematis florida* (Zhou et al., 2024), *Paris polyphylla* var. *Yunnanensis* (Xu B. et al., 2024) and *Pogostemon cablin* (Chen et al., 2024). However, research on *H. japonica* mainly focuses on its chemical composition and pharmacological effects, and there are no reports on the biosynthesis pathway of triterpenoid saponins and its key enzyme genes in *H. japonica.* In this study, RNA-seq was used for the first time to analyze the leaves, roots, and stems of *H. japonica*. The biosynthesis pathway of triterpenoid saponins in *H. japonica* was analyzed at the genetic level. This lays the foundation for the future use of genetic engineering or metabolic engineering techniques to increase the production of triterpenoid saponins and further develop and utilize of triterpenoid saponins in *H. japonica*.

## 2 Materials and methods

### 2.1 Experimental materials

The two-year-old plant of *H. japonica* in this experiment are in vegetative growth stageused and were collected from the herb garden of Anhui University of Chinese Medicine and identified by Professor Qingshan Yang (Supplementary Figure S1). After washing the fresh *H. japonica* with ultrapure water, the tissues of leaves (L), roots (R), and stems (S) were separated and then dried with flter paper. All tissues were quickly frozen in liquid nitrogen.

### 2.2 Extraction of RNA

After high-temperature sterilization of the utensils, the leaves, roots, and stems of *H. japonica* were placed into a mortar and grind them while adding liquid nitrogen. After thorough mixing of the powder, it was placed in a centrifuge tube and the supernatant was collected after centrifugation. The RNA kit (Omega Bio Tek, United States) was used to extract RNA from various tissues. The purity of samples was detected using Thermo NANODROP 2000 ultra micro spectrophotometer, and the concentration and integrity of RNA were detected using Agilent 2,100 bioanalyzer (Supplementary Table S1).

### 2.3 Construction of cDNA library

The extracted RNAs were processed using mRNA enrichment and rRNA removal methods. After the obtained mRNAs were fragmented, the first-strand cDNA and second-strand cDNA were synthesized sequentially. The double-stranded cDNA fragments were subjected to end-repair, and then a single ‘A’ nucleotide was added to the 3′ ends of the blunt fragments. The reaction system and program for adaptor ligation were subsequently configured and set up to ligate adaptors with the cDNAs to obtain the cDNA library.

### 2.4 Transcriptome sequencing and data assembly

The DNA nanoball sequencing (DNB-seq) platform was used for the sequencing of the leaves, roots, and stems of *H. japonica*. Single-stranded circle DNA molecules are replicated via rolling cycle amplification, and a DNB which contain multiple copies of DNA is generated. Sufficient quality DNBs are then loaded into patterned nanoarrays using high-intensity DNA nanochip technique and sequenced through combinatorial Probe-Anchor Synthesis. The SOAPNuke (v1.5.2) software (Cock et al., 2010) was used to filter the raw reads obtained from transcriptome sequencing. After removing reads containing adapters, an unknown base (N) content>10%, and of low quality, clean reads were obtained. After clean reads were assembled using Trinity (v2.0.6) software (Haas et al., 2023), transcripts were clustered and deduplicated using CD-HIT (v4.6) software (Fu et al., 2012) to obtain unigenes.

### 2.5 Functional annotation of unigenes

Unigenes were annotated with seven functional databases using hmmscan (v3.0) software (Johnson et al., 2010), Blast (v2.2.23) software (Altschul et al., 1990), and Blast2GO (v2.5.0) software (Conesa et al., 2005): NCBI Nonredundant Protein Sequence Database (NR), NCBI Nucleotide Database (NT), Manually Annotated and Reviewed Protein Sequence Database (SwissProt), Clusters of Eukaryotic Orthologous Groups Database (KOG), Kyoto Encyclopedia of Genes and Genomes (KEGG), Gene Ontology (GO), Protein Families Database (Pfam). Finally, the functional annotations and classification information corresponding to unigenes were obtained.

### 2.6 Gene expression level and differential expression analysis

Bowtie 2 (v.2.2.5) (Langmead and Salzberg, 2012) and RSEM (v1.2.8) (Li and Dewey, 2011) software were used to calculate the gene expression levels of leaves, roots, and stems of *H. japonica*, and obtained the standard expression level, expressed as fragments per kill of exon model per million mapped fragments (FPKM). Based on the poisson distribution principle, the differentially expressed genes (DEGs) in leaves, roots, and stems of *H. japonica* were analyzed and the functions of DEGs were annotated.

### 2.7 Transcription factor (TF) analysis

The getorf (EMBOSS: 6.5.7.0) and hmmsarch (v3.0) software were used (Rice et al., 2000) to determine the open reading frames (ORFs) of unigenes in *H. japonica*, and compared the ORFs with the TF protein domains. Unigenes were identified based on the TF family characteristics described in PlantTFDB.

### 2.8 Analysis of structural characteristics and phylogenetic of SS

ExPASy (https://web.expasy.org/translate/), MEGA (v5.0) software (Tamura et al., 2011) and CLUSTALX (v1.83) software (Jeanmougin et al., 1998) were used to determine the ORF of SS in *H. japonica* and compare it with the amino acid sequence of SS in *Macleaya cordata*, *Papaver somniferum*, *Glycyrrhiza uralensis*, *Spatholobus suberectus and Glycine max* in the NCBI international database. The secondary and tertiary structures of SS in *H. japonica* were simulated by ESPrip 3.0 (http://esprip.ibcp.fr/ESPri pt/cgi bin/ESPrip.cgi) and Swiss Model (https://swissmodel.epasy.org/), and Pymol (v2.3.2.0) software (Seeliger and de Groot, 2010) were used to visualize its tertiary structure. Phylogenetictrees of SS (CL5764.Contig6) were constructed in MEGA (v5.0) software using the neighbor-joining method with 1,000 bootstrap replicates.

## 3 Results

### 3.1 Transcriptome sequencing and data assembly

The DNB-seq platform was used for transcriptome sequencing of the leaves, roots, and stems of *H. japonica*, and the Q30 of high-quality transcriptome reads were not less than 91.66%. After assembly and redundancy removal, 99,404 unigenes were obtained, with total length, average length, N50, N70, N90, and GC content of 158,559,517 bp, 1595 bp, 2335 bp, 1660 bp, 856 bp, and 39.43%, respectively (Supplementary Table S2). Among the obtained unigenes, 59.82% of the unigenes sequences exceeded 1000 bp, and 43.88% of the unigenes sequences exceeded 1500 bp (Supplementary Figure S2). The benchmarking universal single-copy orthologs was used to evaluate the quality of assembled transcripts, 98% of unigenes were successfully matched, indicating good integrity of transcriptome assembly. The RNAsequencing datasets from leaves, roots, and stems of *H. japonica* were deposited in the NCBI Sequence Read Archive database (accession: PRJNA961922).

### 3.2 Functional annotation and expression level analysis of unigenes

**FIGURE 1:**
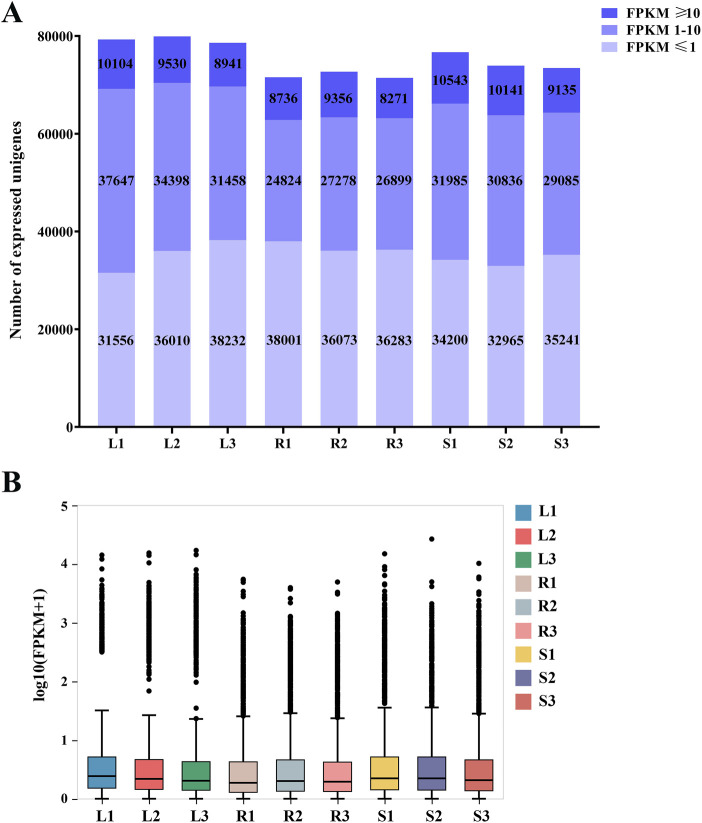
Expression of unigenes in the three tissues of *H. japonica*. **(A)** Distribution of the number of unigenes with different expression levels in the three tissues; **(B)** Boxplots of unigenes expressed in three tissues.

Among the 99,404 unigenes, 7,6071 (76.53%), 59,763 (60.12%), 57,396 (57.74%), 59,788 (60.15%), 60,287 (60.65%), 58,349 (58.70%), and 57,404 (57.75%) were annotated by the seven major functional databases of NR, NT, Swissprot, KOG, KEGG, GO, and Pfam, respectively. 31,061 unigenes were annotated by seven major databases, accounting for 31.25%, while 78,989 unigenes were annotated by any one of the seven databases, accounting for 79.46% (Supplementary Table S3). The expression levels of unigenes in 9 samples of leaves, roots, and stems of *H. japonica* were shown in Figure 1.

### 3.3 Annotations and functional classifcation of unigenes

**FIGURE 2:**
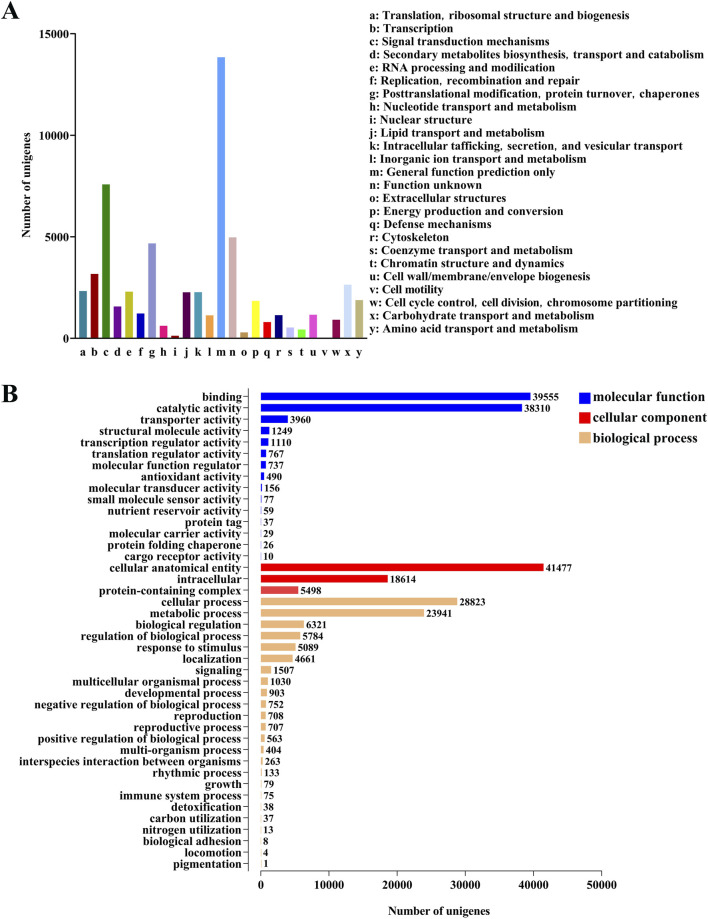
Annotations and functional classification of unigenes in *H. japonica*. **(A)** Gene functional annotation of the KOG database. **(B)** Gene functional annotation of the GO database.

An analysis was conducted on 76,071 unigenes annotated in the NR database. The results showed that in the known gene database, the homology relationship between *M. cordata* (72.28%) and *H. japonica* was the closest, followed by *P. somniferum*, *Nelumbo nucifera*, *Aquilegia coerulea* and *Vitis vinifera* (Supplementary Figuer S3). The KOG database annotated 59,788 unigenes, which were classified into 25 categories (Figure 2A). The main annotations were 13,843 unigenes in “General function prediction only”, 47,848 unigenes in “Signal transduction mechanisms”, and 37,403 unigenes in “Function unknown”.

The unigenes annotated in the NR database were further compared to the GO database, resulting in the annotation of 58,349 unigenes, which were divided into three categories: biological process, cellular component, and molecular function (Figure 2B). The biological process were mainly concentrated in the cellular processes (28,823 unigenes) and the metabolic processes (23,941 unigenes). In terms of cellular composition, it is mainly concentrated in the cellular anatomical entity (41,477 unigenes) and the intracellular (18,614 unigenes). The molecular function mainly focus on the binding (39,555 unigenes) and the catalytic activity (38,310 unigenes).

### 3.4 Analysis of DEGs

**FIGURE 3:**
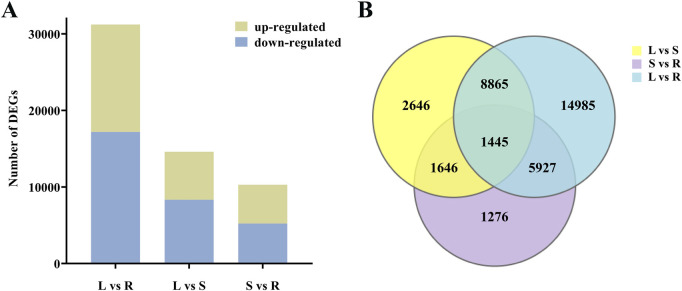
The quantity distribution of DEGs. **(A)** Up-regulated and down-regulated DEGs in different tissues. **(B)** Venn diagram of DEGs in different comparison groups.

In total, 36,790 DEGs were identified between different tissues of *H. japonica*. In the comparison of leaves and roots, a total of 31,222 DEGs were identified, of which 14,040 DEGs were up-regulated and 17,182 DEGs were down-regulated in roots. In the comparison of leaves and stems, a total of 14,602 DEGs were identified, of which 6,268 DEGs were up-regulated and 8,334 DEGs were down-regulated in stems. In the comparison of stems and roots, 10,294 DEGs were identified, of which 5,067 DEGs were up-regulated and 5,227 DEGs were down-regulated in stems (Figure 3A). In addition, we identified 1,445 common DEGs among the three comparisons (Figure 3B).

**FIGURE 4:**
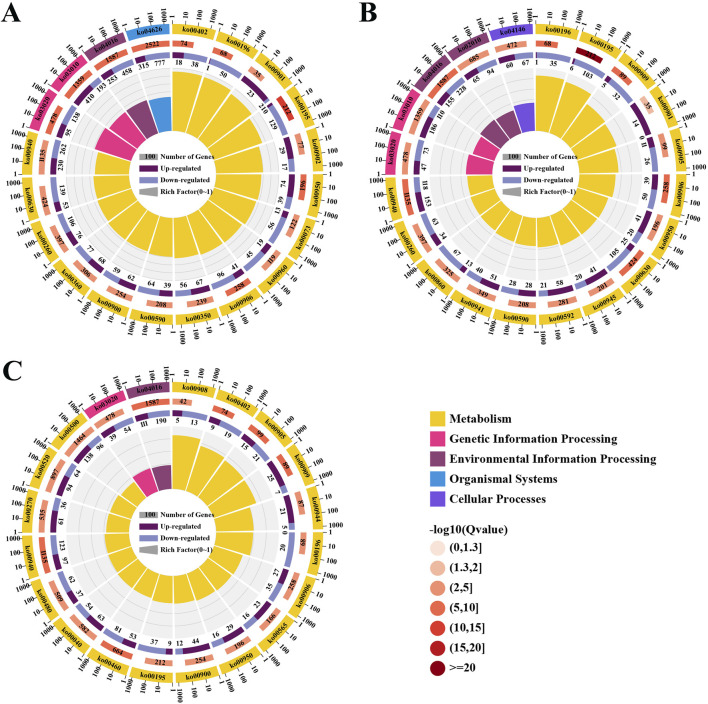
The enrichment analysis of DEGs. **(A)** Enrichment of KEGG pathways for DEGs in the leaves compared to the roots; **(B)** Enrichment of KEGG pathways for DEGs in the stems compared to the leaves; **(C)** Enrichment of KEGG pathways for DEGs in the roots compared to the stems.

The KEGG database was used for biological functional annotation of DEGs. 11,590 DEGs in the comparison of the leaves and roots were annotated to 134 metabolic pathways (Figure 4A), and 5,589 DEGs in the comparison of the stems and leaves were annotated to 134 metabolic pathways (Figure 4B). These two comparisons were mainly enriched in “Plant-pathogen interaction”, “MAPK signaling pathway-plant”, and “Ribosome”. The 154 DEGs compared between leaves and roots involve the biosynthesis pathway of triterpenoid saponins. 80 DEGs are up-regulated in leaves, while 74 DEGs are down-regulated. The 99 DEGs compared between stems and leaves involve the biosynthesis pathway of triterpenoid saponins. 28 DEGs are up-regulated in the stem, while 71 DEGs are down-regulated. 4,091 DEGs in the comparison of the roots and stems were annotated to 133 metabolic pathways, mainly enriched in “Plant-pathogen interaction”, “MAPK signaling pathway-plant” and “Starch and sucrose metabolism” (Figure 4C). A total of 92 DEGs are involved in the biosynthesis pathway of triterpenoid saponins. 59 DEGs are up-regulated in roots, while 33 DEGs are down-regulated.

### 3.5 KEGG enrichment analysis and identification of unigenes related to triterpenoid saponin biosynthesis

**FIGURE 5:**
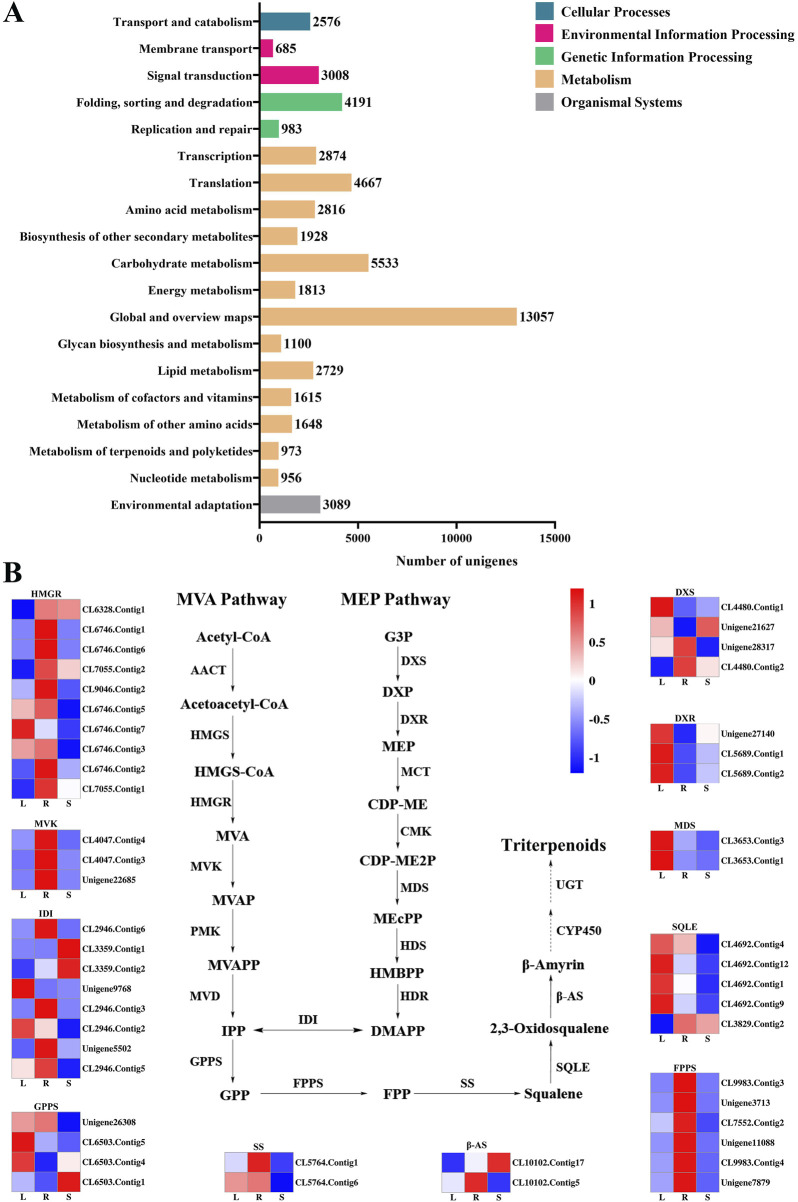
The KEGG annotation of the unigenes in *H. japonica*. **(A)** The KEGG enrichment analysis; **(B)** The expression of key enzyme genes in the biosynthesis pathway of triterpenoid saponins in different tissues.

60,287 unigenes from the transcriptome of *H. japonica* were annotated into the KEGG database, including five categories (Figure 5A): cellular processes (2,576 unigenes), environmental information processing (3,693 unigenes), genetic information processing (12,715 unigenes), metabolism (34,168 unigenes), and organismal systems (3,089 unigenes), involving a total of 135 metabolic pathways. In the metabolic of terpenoids and polyketides, unigenes were mainly distributed in “Carotenoid biosynthesis” (Table 1).

**TABLE 1:** The top 20 metabolic pathways of terpenoids and polyketones.

Pathway name	Pathway ID	Number of unigenes
Carotenoid biosynthesis	ko00906	258
Terpenoid backbone biosynthesis	ko00900	254
Diterpenoid biosynthesis	ko00904	156
Brassinosteroid biosynthesis	ko00905	99
Sesquiterpenoid and triterpenoid biosynthesis	ko00909	89
Monoterpenoid biosynthesis	ko00902	77
Valine, leucine and isoleucine degradation	ko00280	73
Ascorbate and aldarate metabolism	ko00053	73
Tryptophan metabolism	ko00380	70
Fatty acid degradation	ko00071	69
Lysine degradation	ko00310	68
Pyruvate metabolism	ko00620	66
Limonene and pinene degradation	ko00903	64
beta-Alanine metabolism	ko00410	64
Arginine and proline metabolism	ko00330	59
Histidine metabolism	ko00340	58
Glycerolipid metabolism	ko00561	58
Glycolysis/Gluconeogenesis	ko00010	58
MAPK signaling pathway-plant	ko04016	44
Zeatin biosynthesis	ko00908	42

Terpenoid backbone biosynthesis (ko00900) and sesquiterpenoid and triterpenoid biosynthesis (ko00909) were two metabolic pathways involved in the biosynthesis of triterpenoid saponins in *H. japonica*, with a total of 335 unigenes involved. Based on the screening criteria of FPKM>1 of unigenes, there were ten, three, four, three, two, eight, four, and six unigenes for HMGR, MVK, DXS, DXR, MDS, IDI, GPPS, and FPPS in terpenoid skeleton biosynthesis, respectively. In the biosynthetsis pathways of sesquiterpenes and triterpenes, there were two, five, and two unigenes encoding SS, SQLE, and β-AS, respectively (Table 2). The key enzyme unigenes are mostly highly expressed in the leaves and roots of *H. japonica*, while their expression levels were relatively low in the stems. The relative expression levels of these key enzyme unigenes in leaves, roots and stems were displayed in the form of heat maps (Figure 5B).

**TABLE 2:** Key enzymes involved in the biosynthesis pathway of triterpenoid saponins in *H. japonica*.

Name	EC number	Number of genes
hydroxymethylglutaryl-CoA reductase (HMGR)	1.1.1.34	10
mevalonate kinase (MVK)	2.7.1.36	3
1-deoxy-D-xylulose-5-phosphate synthase (DXS)	2.2.1.7	4
1-deoxy-D-xylulose-5-phosphate reductoisomerase (DXR)	1.1.1.267	3
2-C-methyl-D-erythritol 2,4-cyclodiphosphate synthase (MDS)	4.6.1.12	2
isopentenyl-diphosphate delta-isomerase (IDI)	5.3.3.2	8
geranyl diphosphate synthase (GPPS)	2.5.1.1	4
farnesyl diphosphate synthase (FPPS)	2.5.1.10	6
squalene synthase (SS)	2.5.1.21	2
squalene monooxygenase (SQLE)	1.14.14.17	5
beta-amyrin synthase (β-AS)	5.4.99.39	2

### 3.6 TFs involved in the biosynthesis of triterpenoid saponins

**FIGURE 6:**
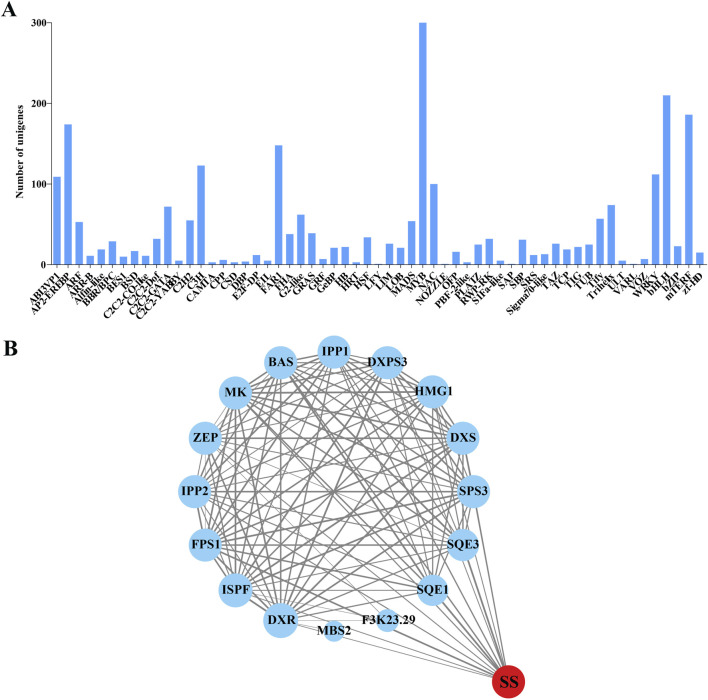
Identification of TFs and the interaction network with key enzymes in the triterpenoid saponins biosynthesis pathway of *H. japonica*. **(A)** Classification of TF families to which isoforms belong. **(B)** Network interactions between key enzymes in the triterpenoid saponins biosynthesis pathway and the TFs family (The size of the circle and the thickness of the line represent the strength of the interaction between proteins).

Based on the transcriptome database of *H. japonica*, 2,550 TFs were identified, belonging to 59 TF families (Figure 6A). The most abundant TF families are MYB (300 unigenes), bHLH (210 unigenes), and mTERF (186 unigenes). According to the functional classification of TFs in the KEGG database, 9 TFs were found to be involved in the metabolism of terpenoids and polyketides. Among them, 4 TFs belong to the FHA family, 3 TFs belong to the ABI3VP1 family, 1 TF belongs to the MYB family, and 1 TF belongs to the bHLH family. Among them, CL3296. Contig9, belonging to the bHLH family, was mainly enriched in terpenoid backbone biosynthesis and was closely related to the biosynthesis pathway of triterpenoid saponins in *H. japonica*. And other TFs were mostly related to the biosynthesis of diterpenes, carotenoids, and secondary metabolites. Protein-protein interaction (PPI) network analysis was performed between these 9 TFs and key enzymes unigenes in the biosynthesis pathway of triterpenoid saponins. The PPI network contained 17 nodes and 107 edges, indicating a high degree of interaction between FPS1, SQE1, and SQE3 with SS (Figure 6B).

### 3.7 Structural characteristics of SS

**FIGURE 7:**
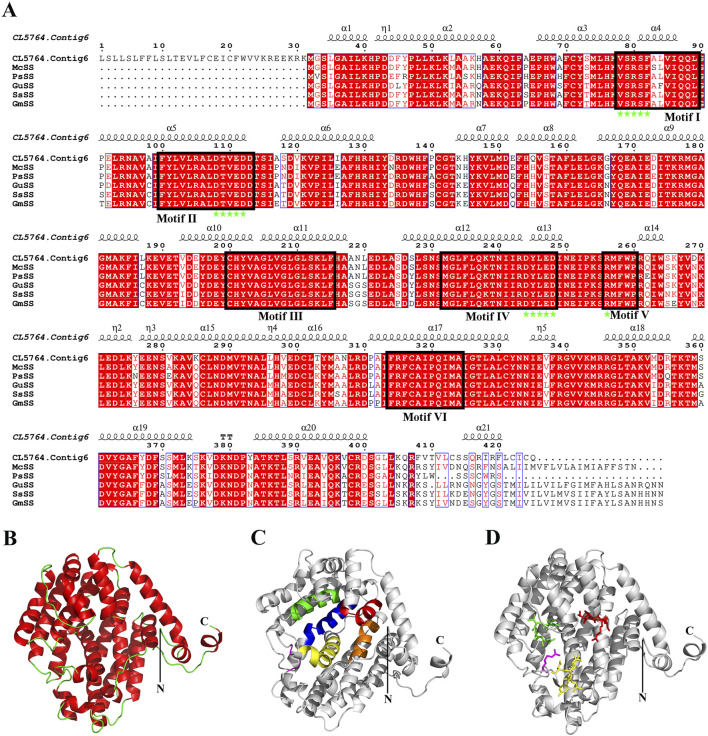
Sequence alignment and protein structure model of SS in *H. japonica*. **(A)** Sequence alignment and secondary structure alignment of SS in *H. japonica* (black box indicating conservative domain Motif I-Motif VI, pentagram indicating four enzyme activity related sites “VSRSF”, “DTVED”, “DYLED” and “R”); **(B–D)** Cartoon model of SS structure in *H. japonica* (red, green, blue, yellow, purple, and brown in Figure C represent the conserved motif I-motif VI, respectively; red, green, yellow, and purple stick models in Figure D represent four enzyme activity related sites “VSRSF”, “DTVED”, “DYLED” and “R”).

**FIGURE 8:**
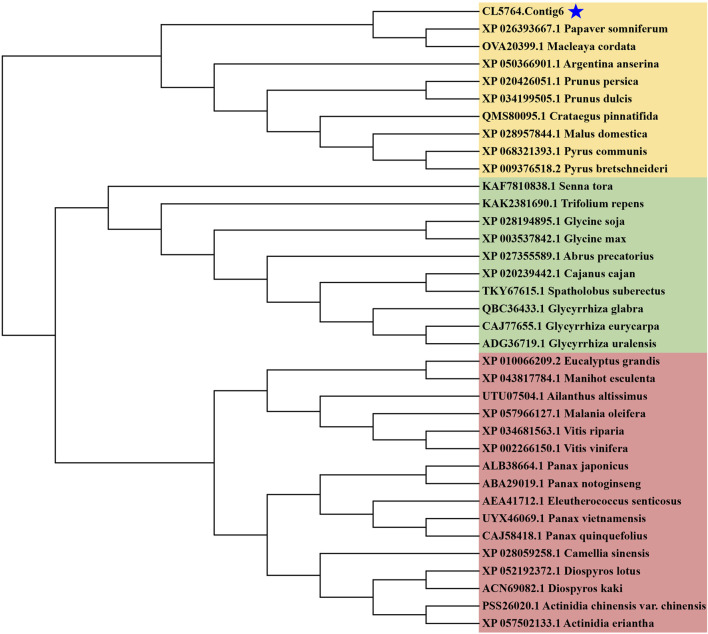
Phylogenetic analysis of SS.

In the biosynthesis pathways of sesquiterpenoid and triterpenoid, Squalene is generated by SS catalyzed FPP, and subsequently forms various conformational changes of triterpenoid saponin aglycones. SS is the first key enzyme in this pathway. The ORF length of CL5764. Contig6 of *H. japonica* was 1278 bp, encoding 425 amino acids. Sequence alignment analysis of SS in *H. japonica* showed 93.01% sequence homology with SS in *M. cordata* (McSS, OVA20399.1), 90.48% sequence homology with SS in *P. somniferum* (PsSS, XP_026393667.1), 88.83% sequence homology with SS in *G. uralensis* (GuSS, ADG36719.1), 88.83% sequence homology with SS in *S. suberectus* (SsSS, TKY67615.1), 88.56% sequence homology with SS in *G. max* (GmSS,NP_001236365.2). The secondary structure of SS in *H. japonica* was mainly composed of α-helices, with a total of 21 α-helices, as well as 1 TT structure and five η structures (Figure 7A). The three-dimensional structure of SS in *H. japonica* constructed by Swiss Model has the highest homology with SS from *P. somniferum* (SMTL ID: A0A4Y7IBE6.1. A), with a similarity of 59%. SS in *H. japonica* contains six conserved regions (Motif I-VI), with four enzyme activity related sites located within the conserved regions (“VSRSF”, “DTFED”, “DYLED” and “R”) (Figures 7B–D) (Tansey and Shechter, 2001). CL5764. Contig6 encoding SS from *H. japonica*, as well as SS from different plant species were chosen to construct a phylogenetic tree through multiple sequence alignment (Figure 8).

## 4 Discussion

Triterpenoid saponins were one of the main active ingredients in *H. japonica* and the material basis for its anti-tumor effects. However, there was a lack of gene level research on the biosynthesis of triterpenoid saponins in *H. japonica.* This study was the first to use DNB-seq technology to sequence the tissues of leaves, roots, and stems of *H. japonica*. After data assembly and redundancy removal, 99,404 unigenes were obtained, with an average length, N50, N70, N90, and the content of GC of 1595 bp, 2335 bp, 1660 bp, 856 bp, and 39.43%, respectively. It indicated that the transcriptome data obtained in this study was reliable and the assembly quality was good.

Previous studies have shown that HMGR, MVK, DXS, DXR, MDS, IDI, GPPS, FPPS, SS, SQLE, and β-AS are key enzymes in the biosynthesis pathway of triterpenoid saponins, playing important roles in the biosynthesis of triterpenoid saponins (Lu et al., 2023; Jiang, 2022; Luo et al., 2016; Xu et al., 2014). After overexpression of HMGR4 and HMGR6 genes in *Arabidopsis thaliana*, the length of the original root was higher than that of the wild type, and the sterol and squalene content significantly increased (Wang Q. et al., 2023). Chen et al. (2017) cloned the MVK gene from *Ginkgo biloba*. After treatment with methyl jasmonate and salicylic acid, the expression level of MVK increased and the downstream product yield increased. Xu et al. (2023) cloned DXS and DXR genes from *G. biloba*, and subcellular localization analysis showed that DXS and DXR1 proteins were located in chloroplasts and cytoplasm, while DXR2 was only located in chloroplasts. Wei et al. (2019) treated *Salvia miltiorrhiza* with inducers and found that the MDS gene was overexpressed in its roots and significantly increased the production of tanshinone. Real time quantitative polymerase chain reaction (RT-qPCR) showed that the expression levels of MEP and MVA pathway genes were positively correlated with increased accumulation of tanshinone. Cloning, expression, and purification of IDI from *Hevea brasiliensis* and *Solanum lycopersicum*, revealed that the enzymes exhibit a complementary sequence, forming additional α-helices around the catalytic site, which can promote biocatalysis (Berthelot et al., 2016). Lan et al. (2024) identified three GPPS genes in flowers of *Osmanthus fragrans*, and transient expression experiments showed that GPPS further improved the biosynthesis of downstream terpenoids. Deng et al. (2022) selected two FPPS genes highly expressed in *H. brasiliensis* to construct prokaryotic expression vectors, and simultaneously performed *in vitro* enzyme activity detection to confirm that FPPS1 and FPPS2 were functional enzymes for natural rubber synthesis, and can directly polymerize IPP and DMAPP to generate FPP. Wang S. et al. (2023) studied the seasonal expression dynamics of key genes related to triterpenoid biosynthesis by RT-qPCR. They found that SQLE and β-AS expression levels were higher in the triterpenoid high content group than in the low content group, and were positively correlated with triterpenoid accumulation. Compared with MVA and MEP pathways in other plants, HMGR, MVK, IDI, and DXS are highly expressed in roots, while DXR and MDS are highly expressed in leaves in *H. japonica*. These results suggest that the MVA and MEP pathways may regulate the synthesis and accumulation of triterpenoid saponins in different tissues of *H. japonica*.

The biosynthesis pathway of triterpenoid saponins in *H. japonica* involves 11 key enzymes and 49 unigenes. The unigenes of HMGR, MVK, IDI, SS, DXS, and FPPS were highly expressed in roots. The unigenes of GPPS, DXR, MDS, and SQLE were highly expressed in leaves, while β-AS gene has the lowest expression level in leaves. SS is the first key enzyme in the pathway of triterpenoid saponin glycoside formation, catalyzing the head-to-tail polymerization of FPP to synthesize squalene (Haralampidis et al., 2002). The SS of *H. japonica* was mainly composed of 21 α-helices, with four enzyme activity sites located in six conserved regions and forming a tubular active center. The structural domains I-VI have substrate or chemical binding sites, Mg^2+^ binding sites, active sites, catalytic domains, regulatory sites, and membrane targeting and anchoring functions, respectively (Wen et al., 2022). The motifs “DTFED” and “DYLED” in the characteristic domains II and IV were aspartic acid (DXXXD) motifs that mediate the binding of isopentenyl diphosphate (Devarenne et al., 2002; Liu et al., 2013).

## 5 Conclusion

In this study, the transcriptome database of the leaves, roots, and stems of *H. japonica* was constructed by the DNB-seq technology. 11 key enzymes and 49 unigenes involved in the biosynthesis pathway of triterpenoid saponins *H. japonica* were identified. In addition, TFs involved in the biosynthesis of triterpenoid saponins in *H. japonica* were also discovered. In summary, this study will contribute to further research on the functional genome of *H. japonica* and provide insights into the biosynthesis mechanism of triterpenoid saponins in *H. japonica*.

## Data Availability

The datasets presented in this study can be found in online repositories. The names of the repository/repositories and accession number(s) can be found below: https://www.ncbi.nlm.nih.gov/, PRJNA961922.
