# Supplementary material for: Comparative transcriptome analysis of different tissues of Hylomecon japonica provides new insights into the biosynthesis pathway of triterpenoid saponins
Source: Front Bioinform. 2025 Jul 7;5:1625145. doi: 10.3389/fbinf.2025.1625145 (PMC12277290; doi:10.3389/fbinf.2025.1625145)
Supplement: Supplementary file 1 [file DataSheet1.doc]

**Supplementary Table S1** Experimental materials and manufacturers*.*

| Reagent | Manufacturer |
| --- | --- |
| DEPC water | Thermo Fisher Scientific Inc. |
| agarose | Thermo Fisher Scientific Inc. |
| Tris-Borate-EDTA Buffer | McLean Chemical Reagent Co., Ltd. |
| ethidium bromide | Xilong Scientific Co., Ltd. |
| chloroform | Xilong Scientific Co., Ltd. |
| isoamyl alcohol | Xilong Scientific Co., Ltd. |
| isopropanol | Xilong Scientific Co., Ltd. |
| β-mercaptoethanol | Sangon Biotech (Shanghai) Co., Ltd. |

**Supplementary Table S2** Quality evaluation of transcriptome data of *H. japonica.*

| Sample | Total Number | Total Length（bp） | Mean Length（bp） | N50（bp） | N70（bp） | N90（bp） | GC(%) |
| --- | --- | --- | --- | --- | --- | --- | --- |
| L1 | 60369 | 75231681 | 1246 | 1874 | 1301 | 610 | 39.73 |
| L2 | 53033 | 64200201 | 1210 | 1829 | 1264 | 582 | 40.01 |
| L3 | 49368 | 59282515 | 1200 | 1801 | 1253 | 583 | 40.15 |
| R1 | 41560 | 48548896 | 1168 | 1735 | 1202 | 565 | 40.63 |
| R2 | 45264 | 55059693 | 1216 | 1825 | 1259 | 588 | 40.38 |
| R3 | 44303 | 53395145 | 1205 | 1798 | 1248 | 584 | 40.31 |
| S1 | 50608 | 61685948 | 1218 | 1803 | 1256 | 601 | 39.99 |
| S2 | 49031 | 64537690 | 1316 | 1996 | 1375 | 646 | 39.94 |
| S3 | 47842 | 61568157 | 1286 | 1949 | 1340 | 623 | 39.96 |
| All unigene | 99404 | 158559517 | 1595 | 2335 | 1660 | 856 | 39.43 |

**Supplementary Table S3** Annotations on the unigenes function of *H. japonica.*

| Database | Number of annotated unigenes | Proportion/% |
| --- | --- | --- |
| NR | 76,071 | 76.53 |
| NT | 59,763 | 60.12 |
| Swissprot | 57,396 | 57.74 |
| KOG | 59,788 | 60.15 |
| KEGG | 60,287 | 60.65 |
| GO | 58,349 | 58.70 |
| Pfam | 57,404 | 57.75 |
| Overall | 78,989 | 79.46 |


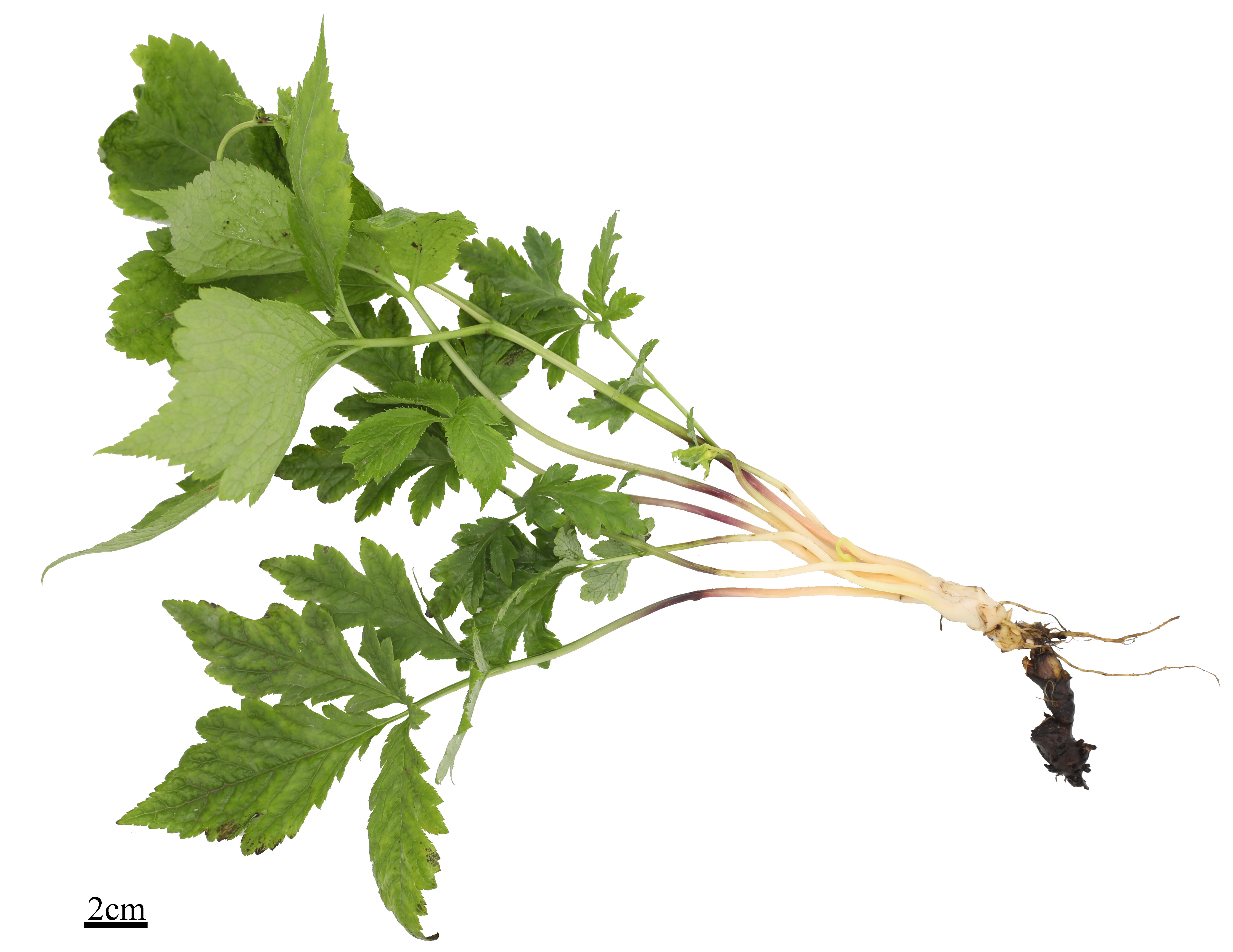


**Supplementary Figure S1** The experiment used *H. japonica* in their vegetative growth stage.


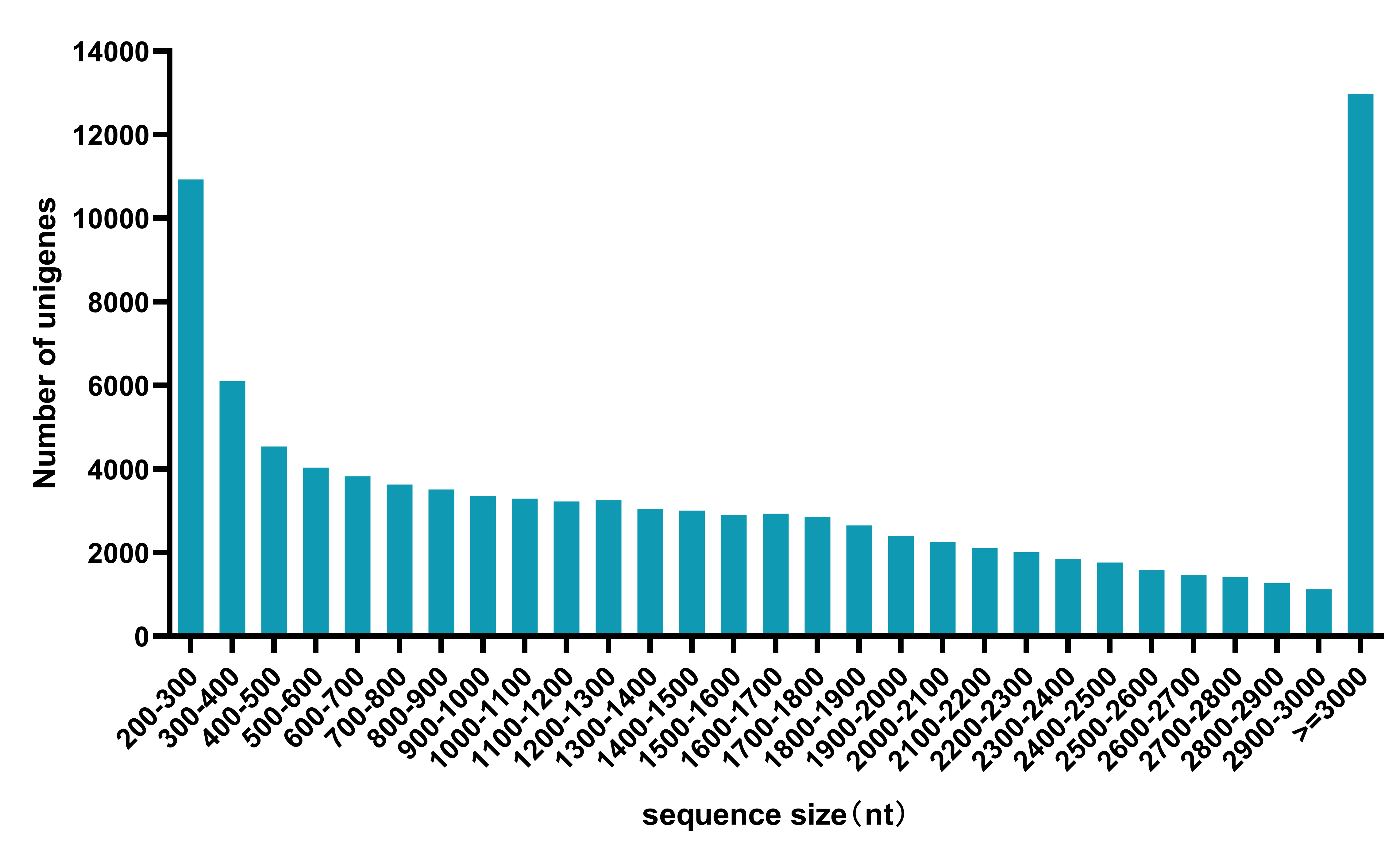


**Supplementary Figure S2** Distribution of unigenes length in *H. japonica.*


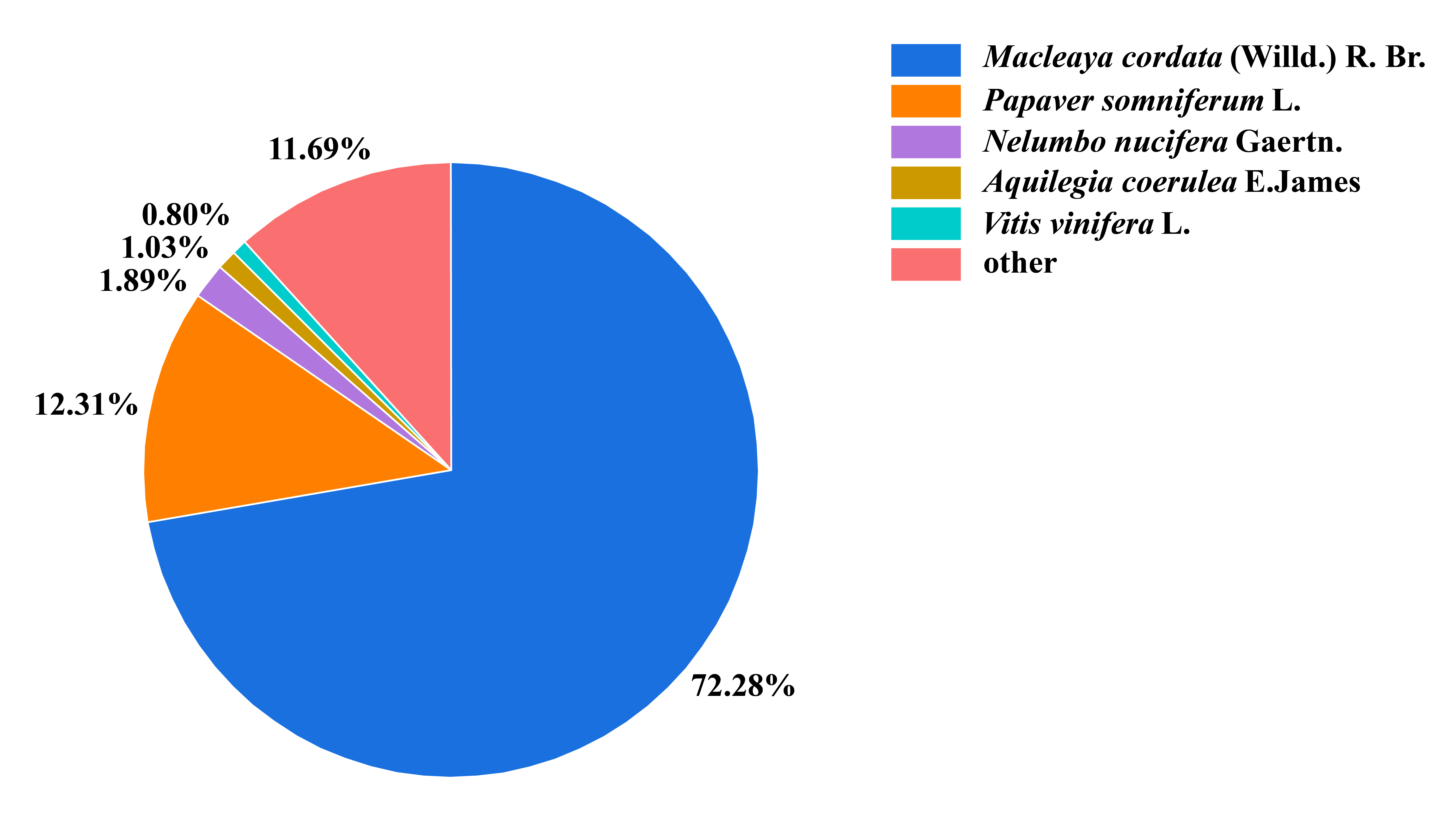


**Supplementary Figure S3** Species distribution of *H. japonica* in NR database.
